# A testing-coverage software reliability model considering fault removal efficiency and error generation

**DOI:** 10.1371/journal.pone.0181524

**Published:** 2017-07-27

**Authors:** Qiuying Li, Hoang Pham

**Affiliations:** 1 School of Reliability & Systems Engineering, Beihang University, Beijing, China; 2 Science & Technology on Reliability & Environmental Engineering Laboratory, Beijing, China; 3 Department of Industrial & Systems Engineering, Rutgers University, Piscataway, New Jersey, United States of America; Universidad de Valladolid, SPAIN

## Abstract

In this paper, we propose a software reliability model that considers not only error generation but also fault removal efficiency combined with testing coverage information based on a nonhomogeneous Poisson process (NHPP). During the past four decades, many software reliability growth models (SRGMs) based on NHPP have been proposed to estimate the software reliability measures, most of which have the same following agreements: 1) it is a common phenomenon that during the testing phase, the fault detection rate always changes; 2) as a result of imperfect debugging, fault removal has been related to a fault re-introduction rate. But there are few SRGMs in the literature that differentiate between fault detection and fault removal, i.e. they seldom consider the imperfect fault removal efficiency. But in practical software developing process, fault removal efficiency cannot always be perfect, i.e. the failures detected might not be removed completely and the original faults might still exist and new faults might be introduced meanwhile, which is referred to as imperfect debugging phenomenon. In this study, a model aiming to incorporate fault introduction rate, fault removal efficiency and testing coverage into software reliability evaluation is developed, using testing coverage to express the fault detection rate and using fault removal efficiency to consider the fault repair. We compare the performance of the proposed model with several existing NHPP SRGMs using three sets of real failure data based on five criteria. The results exhibit that the model can give a better fitting and predictive performance.

## Introduction

Due to software’s ever-increasing usage and crucial role in safety-critical systems, high-quality software products are in great demand. However, the failures of safety-critical software may cause catastrophic loss in life and property. Therefore, software reliability, which is defined as the probability of failure-free software’s operating in a special usage environment for a special period of time [1], has become one of the most important customer-oriented characteristics of software quality. It is very important to have effective approach to develop reliable software along with quantitatively estimating the software reliability [2].

Numerous time-dependent SRGMs have been developed to determine the reliability of software systems [3–7]. These models are usually divided into two categories: one category refers to the perfect debugging model, which assume that each time when a failure occurs, the faults causing the failure are removed instantaneously and no new faults are introduced meanwhile [2, 3, 8]. Most early developed models belong to this category, such as J-M model [9], G-O model [3], delayed S-shaped model [10] and infected S-shaped model [8]. The other category covers the scenario of imperfect debugging, i.e. faults are not always completely eliminated or new faults could be brought in as part of the fault removal process [11–15, 16].

Meanwhile, imperfect debugging can be further divided into two types: one type is that, the fault content of the software is not changed, that is, only the originally detected faults are not removed perfectly without new faults’ birth, i.e. “imperfect fault removal”; on the other hand, in the scenario of another type of imperfect debugging, the total fault content increases as testing progresses because new faults are introduced to the software system while removing the originally detected faults, which is called “error generation”. To sum up, in the case of “imperfect fault removal”, the total fault number of the software is not changed, but in the case of “error generation”, the total fault number keeps increasing in the process of fault removing. It can be clearly seen that, so-called imperfect debugging actually include two meanings, one is aforementioned “error generation”, the other is aforementioned “imperfect fault removal”. But most existing imperfect debugging models only consider “error generation” and neglect “imperfect fault removal”, such as Goel first introduced the conception of imperfect debugging [11], Ohba and Chou proposed an error generation model [17], Pham and Zhang developed an imperfect debugging model with the exponential function of error generation [18], Pham et al. also built a general imperfect debugging model with time-dependent fault content function [19], Yamada gave two imperfect debugging models assuming the fault content function to be exponential or liner function of the testing time, respectively [16].

Nevertheless, it has been often reported that the detected faults are removed with an imperfect removal efficiency instead of 100%. Usually, fault removal efficiency may be less than 100% (e.g., it may range from 15% to 50% for unit testing, 25% to 40% for integration testing, and 25% to 55% for system testing) [20]. Until 2003, Zhang et al. first explicitly proposed a fault removal efficiency model including both “error generation” and “imperfect fault removal” [21]. Kapur et al. explicitly discussed the modeling approach with consideration of the presence of “imperfect debugging” and “error generation” [22]. Goel and Okumoto [23] also developed a similar conception in their Markov model and Kremer [24] proposed a birth-death process to model software reliability, incorporating both imperfect fault removal and fault re-introduction.

Recently, many works have been witnessed in this field of building software reliability models. For example, Wang et al. proposed a comprehensive study to analyze the time dependency between the fault detection and correction processes, they also developed a joint likelihood function for the combined fault detection process and fault correction process to estimate the software reliability model’s parameters [25]. Wang et al. presented an optimal approach repeatedly implementing the function with exponential distribution to fit a logarithmic difference between the estimated values and observed values from a software historical fault data set to improve the software reliability model [26]. Wang et al. applied nonlinear and NHPP imperfect software debugging model in consideration of the fact that the fault introduction is a nonlinear process [27]. Wang et al. developed an imperfect software debugging model considering a log-logistic distribution fault content function, which can capture the increasing and decreasing features of the fault introduction rate [28]. Pham accounted for the uncertainty of operating environments and gave a software reliability model with Vtub-shaped fault-detection rate [29]. Peng et al. studied the fault detection process (FDP) and fault correction process (FCP) with the incorporation of testing effort function and imperfect debugging [30].

However, most of the latest models considered “error generation” neglecting the imperfect fault removal efficiency. Therefore, in-depth and comprehensive consideration of fault removal efficiency is of great importance to build more precise reliability models.

Moreover, researchers have suggested that the accuracy of SRGMs can be further improved by considering the influence of some real issues happening during the testing process [31–33]. Testing coverage is considered as one of the most important factors.

Testing coverage is a good metric for identifying the effectiveness and completeness. Many time-dependent testing coverage functions (TCFs) have been proposed in terms of different distributions, such as Logarithmic-exponential (L-E) [34], S-shaped [35], Rayleigh [36], Weibull & Logistic [37] and Lognormal [38]. Based on different TCFs, software reliability models have also been developed to express the relationship between testing coverage and the cumulative detected faults, such as Beta, Hyper-exponential (H-E) [33], L-E [34], Rayleigh model [36], and some other coverage-based SRGMs [33,35,37].

In this study, we propose a model considering not only error generation, but also imperfect fault removal efficiency incorporating testing coverage. The rest of the paper is organized as follows. In Section 2, we give a brief overview of NHPP and the assumptions for the proposed model, then present the establishment of the proposed model, and several existing SRGMs are also presented. In Section 3, we state two parameter estimation methods and five criteria for models’ performance comparison. In Section 4, we compare the descriptive and predictive performance of this model with several existing NHPP SRGMs on three representative failure data sets, together with the sensitivity analysis. Finally, we draw the conclusions in Section 5.

## Software reliability modeling

### Basic assumptions

The model presented in this paper is based on NHPP, which is utilized to describe the failure phenomenon during the testing phase. The counting process {*N*(*t*), *t* ≥ 0} of NHPP is shown as follows:

$$P\left\{N(t)=i\right\}=\frac{{\left[m(t)\right]}^{i}}{i!}{\text{e}}^{-m(t)},\;i=0,1,2\ldots$$ (1)

The mean value function *m*(*t*) is given as follows:

$$m(t)=\int_{0}^{t}\lambda (u)\text{d}u$$ (2)

where *λ*(*u*) is the fault intensity function.

The proposed model is based on the following assumptions:

1. Software faults’ occurrence and removal follow NHPP.
2. The software failure rate at any time is a function of fault detection rate and the number of faults remaining in the software at that time. The fault detection rate can be expressed by $\frac{c^{\prime }(t)}{1-c(t)}$; *c*(*t*) is the percentage of the code that has been examined up to time *t*, *c*′(*t*) is the derivative of the testing coverage function.
3. When a software failure is detected, an immediate debugging starts, and either (a) the total number of faults is reduced by one with probability *p*, or (b) the total number of faults remains the same with probability 1-*p*.
4. During the fault repair process, whether the fault is removed completely or not, new faults are introduced with a probability constant *α*.

### Model development

Let *c*(*t*) represent the percentage of the code that has been covered up to time *t*. Here c(t) refers to any kind of coverage, e.g. statement coverage, branch coverage, C-use coverage and P-use coverage etc. Obviously, *c*(*t*) is an increasing function of testing time *t*. Usually, it increases very fast from the beginning of software testing process as more test cases are executed to examine the software; after some certain time point, the testing coverage’s increasing rate becomes flat and less because less testing coverage happens to realize the residual fault detection [35]. Thus, a concave or S-shaped function may be used to model the testing coverage function. Apparently, (1-*c*(*t*)) denotes the percentage of the code that has not been examined by test cases up to time *t*. The derivative of testing coverage function, *c*′(*t*), denotes the coverage rate. Thus, the function *c*′(*t*)/(1-*c*(*t*)) is recommended to be used to denote the fault detection rate [35, 37], which has been taken as the common assumption by SRGMs considering testing coverage.

Based on the above assumptions, the mean value function considering both fault removal efficiency and testing coverage can be got by solving the following differential equation:

$$\frac{\text{d}m(t)}{\text{d}t}=\beta \frac{c^{\prime }(t)}{\text{1-}c(t)}[a(t)\text{-}pm(t)]$$ (3)

where *a*(*t*) represents the fault content function of the software, *β* is proportionality constant, *p*is the fault removal efficiency, which means *p*% percentage of detected faults can be removed successfully during the developing process, *m*(*t*) denotes the expected fault number detected up to time *t*, and *pm*(*t*) is the expected fault number that can be eliminated completely, so [*a*(*t*)-*pm*(*t*)] represents the expected remaining fault number presented in the software at time *t*. It should be noted that, when *β* = 1 and *p* ≠ 1, the proposed model has the same form as which is recommended in [21]. When *β* ≠ 1 and *p* = 1, we can get the same form recommended in [22]. Existing models generally assume that *p* equals to 100% [18].

From Assumption 4, the total fault number function *a*(*t*), is a linear function of the expected fault number detected up to time *t*. That is,

a(t)=a+αm(t) (4)

where *a* denotes the initial fault number presented in the software system before testing starts and *α* > 0.

Substituting *a*(*t*) from Eq (4) into Eq (3), and solving it in terms of the initial condition that at *t* = 0, *m*(*t*) = 0, we can obtain

$$m(t)=\frac{a}{p-\alpha }\left[1-{\left(\frac{1-c(0)}{1-c(t)}\right)}^{(\alpha -p)\beta }\right]$$ (5)

where *c*(0) refers to the testing coverage function when *t* = 0.

The software reliability function based on the NHPP is shown as follows:

$$R(x/t)={\text{e}}^{-[m(t+x)-m(t)]}$$ (6)

where *m*(*t*) is given by Eq (5).

### A new model with testing coverage

Substituting different testing coverage function *c*(*t*) into Eq (5), we can get different mean value function *m*(*t*) correspondingly. As mentioned above, the testing coverage’s increasing rate maybe shows a varying trend which firstly increases then decreases. That is, the testing coverage function may show an S-shaped varying trend which is suitable to be described by an S-shaped curve. The inflection S-shaped function is one representative S-shaped function which is flexible and applicable in many cases and has been applied into modeling software reliability [8], so here the following function is used to describe the testing coverage:

$$c(t)=\frac{A(1-{\text{e}}^{-rt})}{1+c{\text{e}}^{-rt}}$$ (7)

where *A* denotes the maximum percentage of testing coverage, *r* is the shape parameter and *c* is the scale parameter. Clearly, when *t* = 0, *c*(0) = 0.

Substituting Eq (7) into Eq (5), we can get the mean value function as follows:

$$m(t)=\frac{a}{p-\alpha }\left[1-{\left(\frac{1+c{\text{e}}^{-rt}}{(1-A)+(c+A){\text{e}}^{-rt}}\right)}^{(\alpha -p)\beta }\right]$$ (8)

It should be noted that both error generation and fault removal efficiency as well as testing coverage are all combined into the proposed model. Table 1 gives a summary of several existing NHPP models and the proposed model.

**Table 1:** Summary of the software reliability models and their mean value functions.

No.	Model name	Mean value function (m(t))
1	G-O model[3]	*m*(*t*) = *a*(1 − e^−*bt*^) *a*(*t*) = *a b*(*t*) = *b*
2	Delayed S-shaped model[10]	*m*(*t*) = *a*(1 − (1 + *bt*)e^−*bt*^) *a*(*t*) = *a* $b(t)=\frac{b^{2}t}{1+bt}$
3	Inflection S-shaped model[8]	$m(t)=\frac{a(1-{\text{e}}^{-bt})}{1+\beta {\text{e}}^{-bt}}$ *a*(*t*) = *a* $b(t)=\frac{b}{1+\beta {\text{e}}^{-bt}}$
4	HD/G-O model[39]	$m(t)=\text{ln}[({\text{e}}^{a}-c)/({\text{e}}^{a{\text{e}}^{-bt}}-c)]$
5	Yamada exponential model[10]	$m(t)=a(1-{\text{e}}^{-\gamma \alpha (1-{\text{e}}^{-\beta t})})$ *a*(*t*) = *a b*(*t*) = *γαβ*e^−*bt*^
6	Yamada Rayleigh model [10]	$m(t)=a(1-{\text{e}}^{-\gamma \alpha (1-{\text{e}}^{-\beta t^{2}/2})})$ *a*(*t*) = *a* $b(t)=\gamma \alpha \beta t{\text{e}}^{-\beta t^{2}/2}$
7	Yamada imperfect 1 model[16]	$m(t)=\frac{ab}{\alpha +b}({\text{e}}^{\alpha t}-{\text{e}}^{-bt})$ *a*(*t*) = *a*e^*αt*^ *b*(*t*) = *b*
8	Yamada imperfect 2 model[16]	$m(t)=a(1-{\text{e}}^{-bt})(1-\frac{\alpha }{b})+\alpha at$ *a*(*t*) = *a*(1 + *αt*) *b*(*t*) = *b*
9	P-Z(1997) model[18]	$m(t)=\frac{1}{\left(1+\beta {\text{e}}^{-bt}\right)}\left(\left(c+a)(1-{\text{e}}^{-bt})-\frac{ab}{b-\alpha }({\text{e}}^{-\alpha t}-e^{-bt}\right)\right)$ *a*(*t*) = *c* + *a*(1 − e^*−αt*^)$b(t)=\frac{b}{1+\beta {\text{e}}^{-bt}}$
10	Fault removal model (2003)[21]	$m(t)=\frac{a}{p-\beta }\left\{1-{\left(\frac{(1+\alpha ){\text{e}}^{-bt}}{\text{1+}\alpha {\text{e}}^{-bt}}\right)}^{\frac{c}{b}(p-\beta )}\right\}$ *a*(*t*) = *βm*(*t*)$b(t)=\frac{c}{1+\alpha {\text{e}}^{-bt}}$
11	SRGM-3 model (2011) [22]	$m(t)=\frac{a}{1-\alpha }\left[1-{\left(\left(1+bt+\frac{b^{2}t^{2}}{2}\right){\text{e}}^{-bt}\right)}^{p(1-\alpha )}\right]$ *a*(*t*) = *a* + *αm*(*t*)$F(t)=1-\left(\sum_{i=0}^{2}\frac{{\left(bt\right)}^{i}}{i!}\right){\text{e}}^{-bt}$
12	Proposed model	$m(t)=\frac{-a}{\alpha -p}\left\{1-{\left(\frac{1+c{\text{e}}^{-rt}}{(1-A)+(c+A){\text{e}}^{-rt}}\right)}^{(\alpha -p)\beta }\right\}$ *a*(*t*) = *a* + *αm*(*t*)$c(t)=\frac{A(1-{\text{e}}^{-rt})}{1+c{\text{e}}^{-rt}}$$b(t)=\frac{c^{\prime }(t)}{1-c(t)}$

## Parameter estimation methods and model comparison criteria

Theoretically, once the analytical expression for *m*(*t*) is derived, the parameters in *m*(*t*) can be estimated by using the maximum likelihood estimation (MLE) method or the least square estimation (LSE) method. MLE is one of the most useful techniques for deriving estimators because comparing to other estimation methods the maximum likelihood estimates are consistent and asymptotically normally distributed as the sample size increases [40]. However, sometimes the estimations may not be obtained by MLE especially under some conditions where *m*(*t*) is too complex, thus we need turn to LSE. So here we use both MLE and LSE methods to estimate the models’ parameters.

### Maximum likelihood estimation (MLE)

Since all the failure data are expressed in the form of pairs (*t*_*i*_, *y*_*i*_) (*i* = 1,2,…, *n*; 0 < *t*_1_ < *t*_2_ < ⋯ < *t*_*n*_), where *y*_*i*_ is the cumulative number of faults detected in time (0, *t*_*i*_], basing on the definition of NHPP, the likelihood function is given as follows:

$$L=\prod_{i=1}^{n}\frac{{\left(m(t_{i})\text{-}m(t_{i\text{-1}})\right)}^{y_{i}\text{-}y_{i\text{-}1}}}{(y_{i}\text{-}y_{i\text{-1}})!}e^{\text{-}(m(t_{i})\text{-}m(t_{i\text{-1}}))}$$ (9)

The logarithmic form of the above likelihood function is given as follows:

$$\text{ln}L=\sum_{i=1}^{n}\left\{(y_{i}\text{-}y_{i\text{-1}})\text{ln}(m(t_{i})\text{-}m(t_{i\text{-1}})\right)\text{-}(m(t_{i})\text{-}m(t_{i\text{-1}}))\text{-ln(}(y_{i}\text{-}y_{i\text{-1}})!\text{)}\}$$ (10)

By taking derivatives of Eq (10) with respect to each parameter in *m*(*t*), and setting the results equal to zero, we can obtain equations for the proposed model as follows:

$$\frac{\partial \text{ln}L}{\partial a}=\frac{\partial \text{ln}L}{\partial \alpha }=\frac{\partial \text{ln}L}{\partial p}=\frac{\partial \text{ln}L}{\partial \beta }=\frac{\partial \text{ln}L}{\partial c}=\frac{\partial \text{ln}L}{\partial r}=\frac{\partial \text{ln}L}{\partial A}=0$$ (11)

After solving the above equations simultaneously, we can obtain the maximum likelihood estimates of all parameters for the proposed model.

### Least square estimation (LSE)

LSE is based on minimizing the sum of the squared distance from the best fit line and the actual data points. The sum of the squared distance is given as follows:

$$Q=\sum_{i=1}^{n}{\left(y_{i}\text{-}m(t_{i})\right)}^{2}$$ (12)

By taking derivatives of Eq (12) with respect to each parameter in *m*(*t*), and setting the results equal to zero, we can obtain equations for the proposed model as follows:

$$\frac{\partial Q}{\partial a}=\frac{\partial Q}{\partial \alpha }=\frac{\partial Q}{\partial p}=\frac{\partial Q}{\partial \beta }=\frac{\partial Q}{\partial c}=\frac{\partial Q}{\partial r}=\frac{\partial Q}{\partial A}=0$$ (13)

After solving the above equations simultaneously, we can obtain the least square estimates of all parameters for the proposed model.

### Criteria for models’ descriptive power comparison

Here we use four criteria to examine the descriptive performance of SRGMs. The first criterion is the mean value of squared error (Mean Square-Error, MSE), which is defined as follows [41]:

$$\text{MSE}=\frac{1}{n-N}\sum_{i=1}^{n}{\left(y_{i}-\hat{m}(t_{i})\right)}^{2}$$ (14)

where *n* is the number of observations, $\hat{m}(t_{i})$ is the estimated value of cumulative fault number up to time *t*_*i*_ according to the fitted mean value function, *i* = 1,2,…,*n*. *N* represents the number of parameters used in the model.

In practice, when comparing the performance of models with different numbers of parameters, it is always considered unfair to simply compare the performance of models owning more parameters with others owning fewer parameters without giving any penalty to those models with more parameters. So it should be noted that here MSE value considers the penalty term with respect to the degrees of freedom when there are many parameters and assigns a larger penalty to a model with more parameters. Thus, more parameters, more penalty will be given; so lower value of MSE indicates better goodness of fit.

The second criterion which is used to examine the fitting power of SRGMs is correlation index of the regression curve equation (*R*^2^), which is expressed as follows:

$$R^{2}=1-\frac{\sum_{i=1}^{n}{\left(y_{i}-\hat{m}(t_{i})\right)}^{2}}{\sum_{i=1}^{n}{\left(y_{i}-\bar{y}\right)}^{2}}$$ (15)

where $\bar{y}=\frac{1}{n}\sum_{i=1}^{n}y_{i}$. Therefore, the more *R*^2^, the better is the model’s performance.

The third criterion which is used to evaluate the performance of SRGMs is adjusted *R*^2^ (Adjusted_*R*^2^), which can be expressed as follows:

$$\text{Adjusted\_}R^{2}\;=1-\frac{\left(1-R^{2})(n-1\right)}{n-P-1}$$ (16)

where the value of *R*^2^ in the right side of Eq (16)is shown as Eq (15) and *P* represents the number of predictors in the fitted model. Therefore, the more Adjusted_*R*^2^, the better is the model’s goodness-of-fit. Here the penalty with respect to the number of model’s parameter is also considered.

The four criterion is AIC, which measures the ability of a model to maximize the likelihood function that is directly related to the degrees of freedom during fitting and defined as follows:

AIC=−2logL+2N (17)

where *L* is the value of likelihood function at its maximum, *N* represents the number of parameters used in the model. The lower value of AIC indicates better goodness-of-fit.

It should be noted that AIC takes the degrees of freedom into consideration by assigning a larger penalty to a model with more parameters. The number of parameters are also considered in MSE and Adjusted_*R*^2^, where a larger penalty will be assigned to a model with more parameters.

### Criteria for models’ predictive power comparison

Here we use SSE criterion to examine the predictive power of SRGMs. SSE is the sum of squared error, which is expressed as follows [42]:

$$\text{SSE}=\sum_{i=m}^{n}{\left(y_{i}-\hat{m}(t_{i})\right)}^{2}$$ (18)

Assume that by the end of testing time *t*_*n*_, totally *y*_*n*_ faults have been detected. Firstly we use the data points up to time *t*_*m*-1_(*t*_*m*-1_ < *t*_*n*_) to estimate the parameters of *m*(*t*), then substituting the estimated parameters in the mean value function yields the prediction value of the cumulative fault number $\hat{m}(t_{m})$ by *t*_*m*_ (*t*_*m*_ < *t*_*n*_), *y*_*m*_ is the actual number of faults detected by *t*_*m*_. Then the procedure is repeated for several values of *t*_*i*_ (*i* = *m* + 1, *m* + 2, … *n*.) until *t*_*n*_.

Therefore, the less SSE, the better is the model’s performance.

## Model analysis with real application

Here we examine the performance of the proposed model compared to several existing NHPP models basing on three representative data sets.

### Monitor and control system data

The first data set is large in size and collected from testing a real monitor and control system (Data Set 1, DS-1) [43], which has been widely used in many studies, such as [42]. The details are recorded in Table 2 and the time unit is day. There are totally 481 faults observed within 111 days. All data points are used to fit the models and estimate the models’ parameters.

**Table 2:** Failure data of DS-1.

Day No.	Cumulative faults	Day No.	Cumulative faults	Day No.	Cumulative faults	Day No.	Cumulative faults
1	5	29	254	57	448	85	473
2	10	30	259	58	451	86	473
3	15	31	263	59	453	87	475
4	20	32	264	60	460	88	475
5	26	33	268	61	463	89	475
6	34	34	271	62	463	90	475
7	36	35	277	63	464	91	475
8	43	36	290	64	464	92	475
9	47	37	309	65	465	93	475
10	49	38	324	66	465	94	475
11	80	39	331	67	465	95	475
12	84	40	346	68	466	96	476
13	108	41	367	69	467	97	476
14	157	42	375	70	467	98	476
15	171	43	381	71	467	99	476
16	183	44	401	72	468	100	477
17	191	45	411	73	469	101	477
18	200	46	414	74	469	102	477
19	204	47	417	75	469	103	478
20	211	48	425	76	469	104	478
21	217	49	430	77	470	105	478
22	226	50	431	78	472	106	479
23	230	51	433	79	472	107	479
24	234	52	435	80	473	108	479
25	236	53	437	81	473	109	480
26	240	54	444	82	473	110	480
27	243	55	446	83	473	111	481
28	252	56	446	84	473		

Here the model parameters estimated by both LSE and MLE are given in Table 3, respectively. MSE values, *R*^2^ values and Adjusted *R*^2^ values are obtained basing on the parameters estimated by LSE method and AIC values are given basing on parameters estimated by MLE method.

**Table 3:** Comparison of SRGMs’ descriptive power for DS-1.

Model No.	Model Name	LSE Method				MLE Method	
		Model Parameter Estimation Results	MSE	*R*^2^	Adjusted_ *R*^2^	Model Parameter Estimation Results	AIC
1	G-O model	$\hat{a}=538.1$ $\hat{b}=0.02575$	804.2202	0.9646	0.9643	$\hat{a}=497.3$ $\hat{b}=0.03080$	723.7554
2	Delayed S-shaped model	$\hat{a}=488.1$ $\hat{b}=0.06629$	331.8349	0.9854	0.9853	$\hat{a}=483.0$ $\hat{b}=0.06865$	644.0284
3	Inflection S-shaped model	$\hat{a}=484.6$ $\hat{b}=0.06681$ $\hat{\beta }=3.648$	300	0.9869	0.9867	$\hat{a}=482.0$ $\hat{b}=0.07021$ $\hat{\beta }=4.146$	**641.8546**
4	HD/G-O model	$\hat{a}=538.1$ $\hat{b}=0.02575$ $\hat{c}=2.849$	811.6667	0.9646	0.9639	$\hat{a}=\text{497}.3$ $\hat{b}=\text{0}.03080$ $\hat{c}=\text{0}.09586$	725.7554
5	Yamada exponential model	$\hat{a}=6.987\text{e}+04$ $\hat{\beta }=0.02566$ $\hat{\gamma }=0.03822$ $\hat{\alpha }=0.2023$	820.9346	0.9645	0.9635	$\hat{a}=\text{4}.974\text{e}+02$ $\hat{\beta }=\text{1}.167\text{e}-05$ $\hat{\gamma }=\text{31}.19$ $\hat{\alpha }=\text{84}.55$	727.8002
6	Yamada Rayleigh model	$\hat{a}=566$ $\hat{\beta }=0.001065$ $\hat{\gamma }=1.463$ $\hat{\alpha }=1.269$	461.5888	0.98	0.9795	$\hat{a}=\text{499}.4$ $\hat{\beta }=6.53\text{e}-04$ $\hat{\gamma }=0.2465$ $\hat{\alpha }=13.628$	669.3596
7	Yamada imperfect 1 model	$\hat{a}=538.1$ $\hat{b}=0.02575$ $\hat{\alpha }=4.124\text{e}-10$	811.6667	0.9646	0.9643	$\hat{a}=\text{497}.3$ $\hat{b}=\text{0}.03080$ $\hat{\alpha }=\text{0}$	725.7554
8	Yamada imperfect 2 mode	$\hat{a}=538.1$ $\hat{b}=0.02575$ $\hat{\alpha }=1.074\text{e}-12$	811.6667	0.9646	0.9643	$\hat{a}=\text{497}.3$ $\hat{b}=\text{0}.03080$ $\hat{\alpha }=\text{0}$	725.7554
9	P-Z model	$\hat{a}=0.9988$ $\hat{b}=0.06682$ $\hat{c}=483.6$ $\hat{\alpha }=0.5211$ $\hat{\beta }=3.65$	305.6604	0.9869	0.9864	$\hat{a}=1.0$ $\hat{b}=\text{0}.07021$ $\hat{c}=481.0$ $\hat{\alpha }=5.3749$ $\hat{\beta }=4.146$	645.8532
10	Fault removal model	$\hat{a}=115$ $\hat{\alpha }=0.9999$ $\hat{b}=0.07023$ $\hat{p}=0.6158$ $\hat{c}=0.1698$ $\hat{\beta }=0.3886$	477.4286	0.9797	0.9788	$\hat{a}=300.0$ $\hat{\alpha }=4.3958$ $\hat{b}=0.06336$ $\hat{p}=0.8597$ $\hat{c}=0.1229$ $\hat{\beta }=0.2369$	647.5668
11	SRGM-3 model	$\hat{A}=480.7$ $\hat{\alpha }=0.02476$ $\hat{b}=0.3128$ $\hat{p}=0.1695$	354.8598	0.9847	0.9842	$\hat{a}=400.0$ $\hat{\alpha }=0.1748$ $\hat{b}=0.3643$ $\hat{p}=0.1753$	662.7954
12	proposed model	$\hat{a}=408$ $\hat{\alpha }=0.2301$ $\hat{c}=0.7125$ $\hat{p}=0.6443$ $\hat{\beta }=0.1726$ $\hat{\gamma }=0.1703$ $\hat{A}=0.9999$	**239.4231**	**0.9899**	**0.9894**	$\hat{a}=108.3$ $\hat{\alpha }=0.4548$ $\hat{c}=1.1297$ $\hat{p}=0.5199$ $\hat{\beta }=1.5890$ $\hat{\gamma }=0.0883$ $\hat{A}=0.9633$	647.31

Notes: The bold numbers mean the results of the best SRGM in this column.

It can be seen that compared to all models using MSE, *R*^2^ and Adjusted *R*^2^ criteria, the proposed model displays the smallest MSE value, the largest *R*^2^ value and Adjusted_ *R*^2^ value at 239.4231, 0.9899 and 0.9894, respectively. Although the inflection S-shaped model also fits well, its values are still larger or smaller than those of the proposed model. Though the AIC value of the proposed model is not the smallest one among all models, that is, the value of AIC at 647.31 is a little bigger than those values of the inflection S-shaped model, the delayed S-shaped and the P-Z model. But this value is not very bigger than the smallest value of the inflection S-shaped model at 641.8546, the value of the delayed S-shaped model at 644.0284 and P-Z model’s value of 645.8532, as well very less than the value of the Yamada exponential model at 727.8002. So we can deduce that the descriptive power of our proposed model is better than those of other models.

Moreover, some additional information can be acquired from the estimation values of the parameters given by the proposed model. For instance, in the context of LSE method, the fault removal efficiency is 64.43%, which is less than the average value according to [20] (The range of the fault removal efficiency was from 45% to 99% with the average value of 72%). Therefore, more resources should be allocated to enhance the efficiency of the fault removal. Moreover, the fault removal probability on per failure is 0.6443 which is a lower value, and the fault introduction rate is 0.2301 which is not a very low value. That means if those models neglecting imperfect debugging are used, more deviation will be introduced. The initial fault content is estimated to be 408, together with 0.2301 fault introduction rate and 481 total detected faults, then the expected number of total detected faults is 519. Thus, at 111 days, which is the assumed stopping time point, there are still 38 faults latent in the software. The fault introduction rate is 0.2301, i.e., 1 fault will be introduced when 4 faults are removed on average. So more testing training should be given to the testers and their testing skill should be improved greatly.

**Fig 1:**
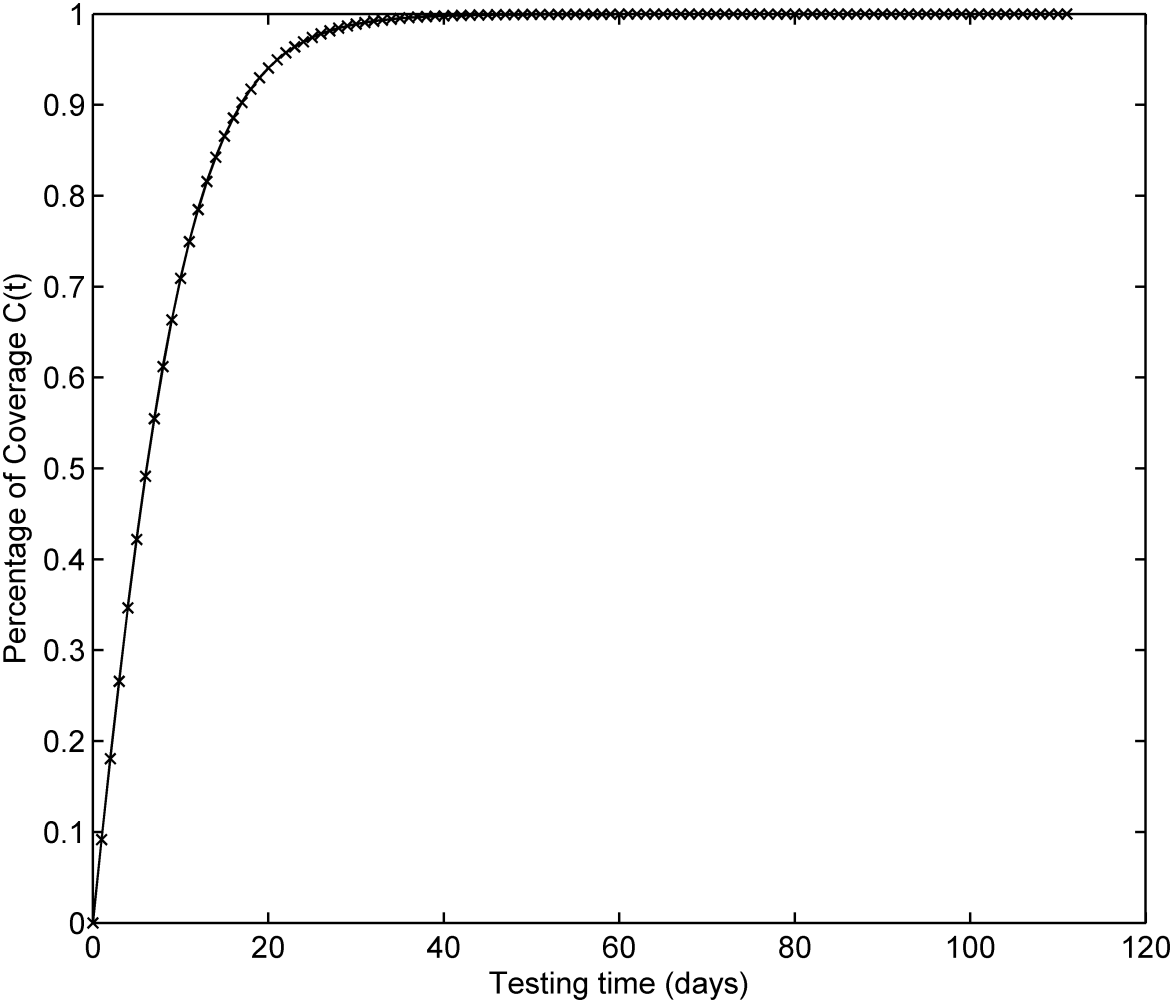
Testing coverage of DS-1.

**Fig 2:**
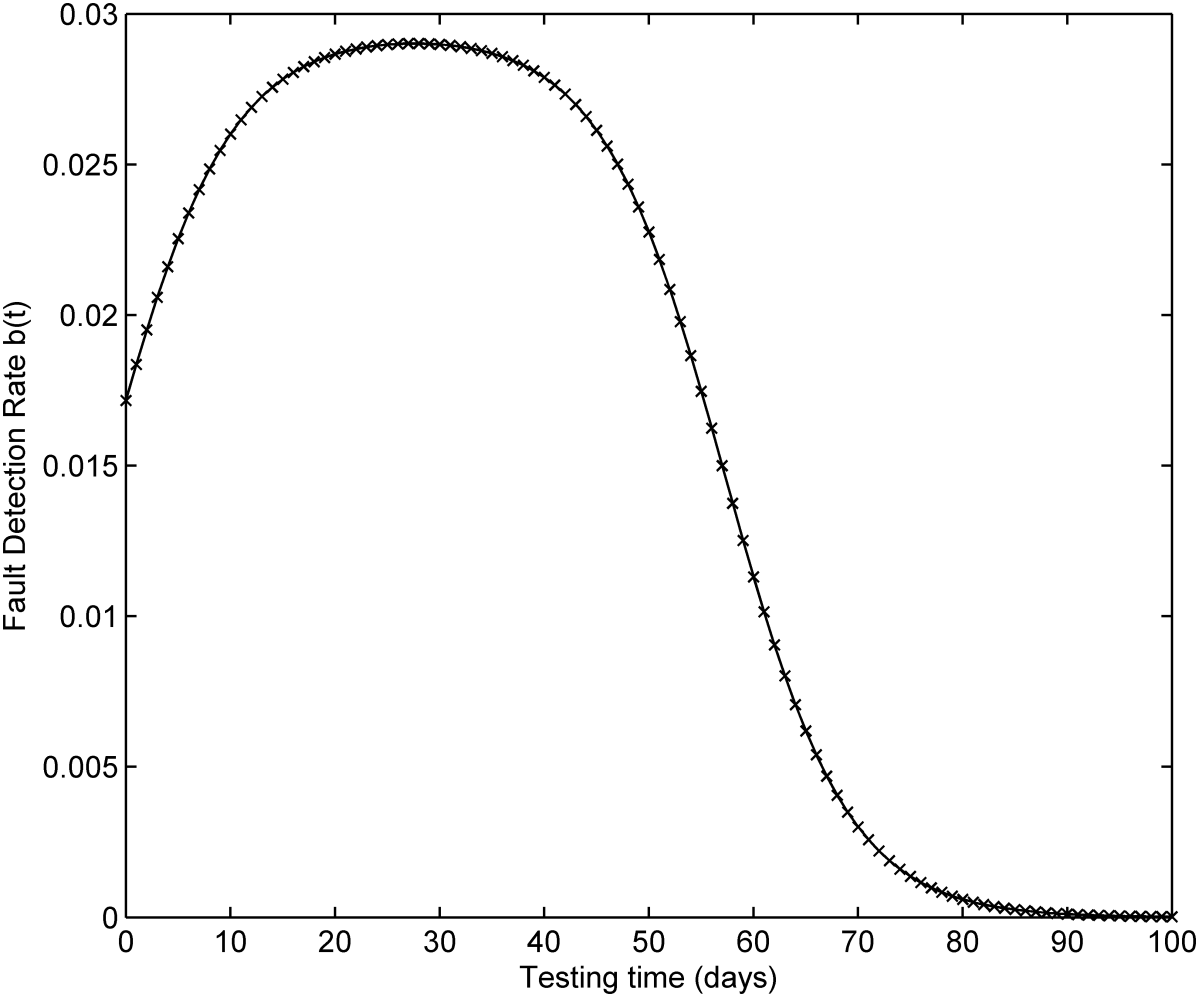
Fault detection rate of DS-1.

**Fig 3:**
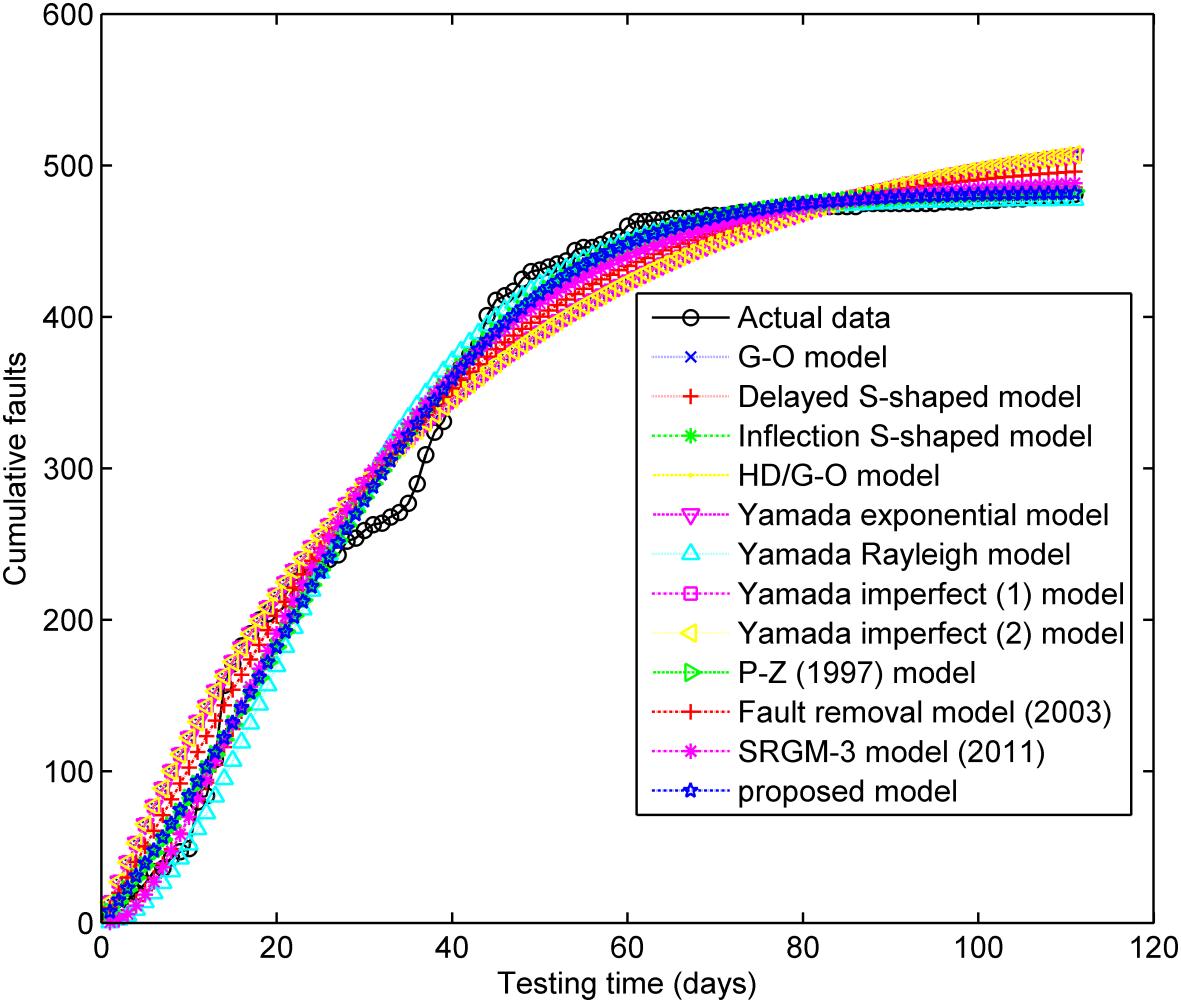
The fitting results of SRGMs compared with actual data for DS-1.

Fig 1 depicts the testing coverage function of the proposed model based on the parameters estimated by LSE according to DS-1, the changing trend of the testing coverage over time shows the aforementioned trend, i.e. first it increases very fast at the beginning of testing process, then after some certain time point, the testing coverage’s increasing rate becomes flat and less. Fig 2 shows the fault detection rate over time of DS-1. It is clearly shown that the fault detection rate shows a varying trend which firstly increases and then decreases with an S-shaped varying trend. The fitting comparison of all models for DS-1 is graphically illustrated in Fig 3. In terms of Fig 3, it can be seen that the proposed model fits the actual data better than all other models.

### Tandem computer data

In this section, we examine models using another data collected from Tandem Computers Release #1 (Data Set 2, DS-2) [2], which has also been widely used in many studies, such as [7,42,44]. DS-2 is small in size and the failure data are tabulated in Table 4 with time unit week. There are totally 100 faults detected within about 20 weeks. All data points are used to fit the models and estimate the models’ parameters.

**Table 4:** Failure data of DS-2.

Testing time (weeks)	CPU (hours)	Cumulative faults	Testing time (weeks)	CPU (hours)	Cumulative faults
1	519	16	11	6539	81
2	968	24	12	7083	86
3	1430	27	13	7487	90
4	1893	33	14	7846	93
5	2490	41	15	8205	96
6	3058	49	16	8564	98
7	3625	54	17	8923	99
8	4422	58	18	9282	100
9	5218	69	19	9641	100
10	5823	75	20	10000	100

As the same as in the first case study, we also use both LSE and MLE to estimate the models’ parameters recorded in Table 5. MSE, *R*^2^ and Adjusted *R*^2^ values are calculated on the parameters obtained by LSE method and AIC values in the context of MLE method.

**Table 5:** Comparison of SRGMs’ descriptive power for DS-2.

Model No.	Model Name	LSE Method				MLE Method	
		Model Parameter Estimation Results	MSE	*R*^2^	Adjusted_ *R*^2^	Model Parameter Estimation Results	AIC
1	G-O model	$\hat{a}=130.2$ $\hat{b}=0.08317$	12.9056	0.9857	0.9849	$\hat{a}=112.5$ $\hat{b}=0.1099$	89.70326
2	Delayed S-shaped model	$\hat{a}=104$ $\hat{b}=0.2654$	28.0611	0.9689	0.9672	$\hat{a}=102.3$ $\hat{b}=0.285$	110.5186
3	Inflection S-shaped model	$\hat{a}=110.8$ $\hat{b}=0.1721$ $\hat{\beta }=1.205$	10.5647	0.989	0.9877	$\hat{a}=104.2$ $\hat{b}=0.2034$ $\hat{\beta }=1.4366$	**89.11096**
4	HD/G-O model	$\hat{a}=130.2$ $\hat{b}=0.08317$ $\hat{c}=0.2769$	13.6647	0.9857	0.984	$\hat{a}=112.5$ $\hat{b}=0.1099$ $\hat{c}=1.0\text{e-8}$	91.70326
5	Yamada exponential model	$\hat{a}=999.5$ $\hat{\beta }=0.07685$ $\hat{\gamma }=0.279$ $\hat{\alpha }=0.51$	14.8438	0.9854	0.9827	$\hat{a}=112.5$ $\hat{\beta }=\text{1}.104\text{e}-04$ $\hat{\gamma }=38.83$ $\hat{\alpha }=25.61$	93.7099
6	Yamada Rayleigh model	$\hat{a}=115.8$ $\hat{\beta }=0.01721$ $\hat{\gamma }=3.03$ $\hat{\alpha }=0.6548$	49.4188	0.9514	0.9422	$\hat{a}=127.7$ $\hat{\beta }=0.02003$ $\hat{\gamma }=3.31$ $\hat{\alpha }=0.4708$	128.5853
7	Yamada imperfect 1 model	$\hat{a}=130.2$ $\hat{b}=0.08317$ $\hat{\alpha }=9.363\text{e}-10$	13.6647	0.9857	0.9849	$\hat{a}=112.5$ $\hat{b}=0.1099$ $\hat{\alpha }=1.0\text{e-9}$	91.70326
8	Yamada imperfect 2 mode	$\hat{a}=130.2$ $\hat{b}=0.08317$ $\hat{\alpha }=1.04\text{e}-09$	13.6647	0.9857	0.9849	$\hat{a}=112.5$ $\hat{b}=0.1099$ $\hat{\alpha }=1.0\text{e-10}$	91.70326
9	P-Z model	$\hat{a}=1.589\text{e}-08$ $\hat{b}=0.1721$ $\hat{c}=110.8$ $\hat{\alpha }=0.000368$ $\hat{\beta }=1.205$	11.9733	0.989	0.986	$\hat{a}=1.0\text{e-10}$ $\hat{b}=0.2034$ $\hat{c}=104.2$ $\hat{\alpha }=2.415$ $\hat{\beta }=1.437$	93.11096
10	Fault removal model	$\hat{a}=110$ $\hat{\alpha }=1$ $\hat{b}=0.1446$ $\hat{p}=0.9945$ $\hat{c}=0.1833$ $\hat{\beta }=0.1064$	12.8929	0.9889	0.9849	$\hat{a}=83.1$ $\hat{\alpha }=100$ $\hat{b}=0.1066$ $\hat{p}=0.8826$ $\hat{c}=9.9982$ $\hat{\beta }=0.0549$	91.34258
11	SRGM-3 model	$\hat{A}=130.8$ $\hat{\alpha }=0.01157$ $\hat{b}=1$ $\hat{p}=0.2277$	37.6375	0.963	0.9586	$\hat{a}=80$ $\hat{\alpha }=0.2803$ $\hat{b}=37.25$ $\hat{p}=0.0044$	95.25002
12	proposed model	$\hat{a}=59$ $\hat{\alpha }=0.5148$ $\hat{c}=8.19\text{e-05}$ $\hat{p}=0.8248$ $\hat{\beta }=0.2594$ $\hat{\gamma }=0.6189$ $\hat{\text{A}}=0.9999$	**8.8385**	**0.9929**	**0.9897**	$\hat{a}=58.3$ $\hat{\alpha }=0.5389$ $\hat{c}=1.0\text{e-06}$ $\hat{p}=0.8222$ $\hat{\beta }=0.2557$ $\hat{\gamma }=0.6369$ $\hat{\text{A}}=0.9999$	90.4466

Notes: The bold numbers mean the result of the best SRGM in this column.

It can be seen that compared to all models using the MSE, *R*^2^ and Adjusted *R*^2^ criteria, the proposed model displays the smallest MSE value, the largest *R*^2^ value and Adjusted_*R*^2^ value, the values are 8.8385, 0.9929 and 0.9897, respectively. Although the inflection S-shaped model also fits well, its values 10.5647, 0.989, 0.9877 are still larger or smaller than those of the proposed model. Though the AIC value of the proposed model is not the smallest one compared to the ones of the inflection S-shaped model and G-O model, it is still not very bigger than them and much smaller than other models’ values. So we can deduce that the proposed model performs comparably better than those of other models in goodness-of-fit behavior.

Moreover, some additional information can be acquired from the estimation values of the parameters given by the proposed model. For instance, in the context of LSE method, the fault removal efficiency is 82.48%, which is slightly higher than the average value according to [20], indicating the skill level of the testing team is beyond the average level. The initial fault content is estimated 59, and the fault introduction rate is 0.5148, the expected total number of faults detected is 110. Thus, at 20 weeks, which is the assumed stopping time point, there are still about 10 remaining faults present in the software. This means that some faults still remain in the software at the end of the testing phase. The fault introduction probability is 0.5148, i.e., on average, 1 fault will be introduced when 2 faults are removed on average. So more testing training should be given to the testers and their testing skill should be improved greatly.

**Fig 4:**
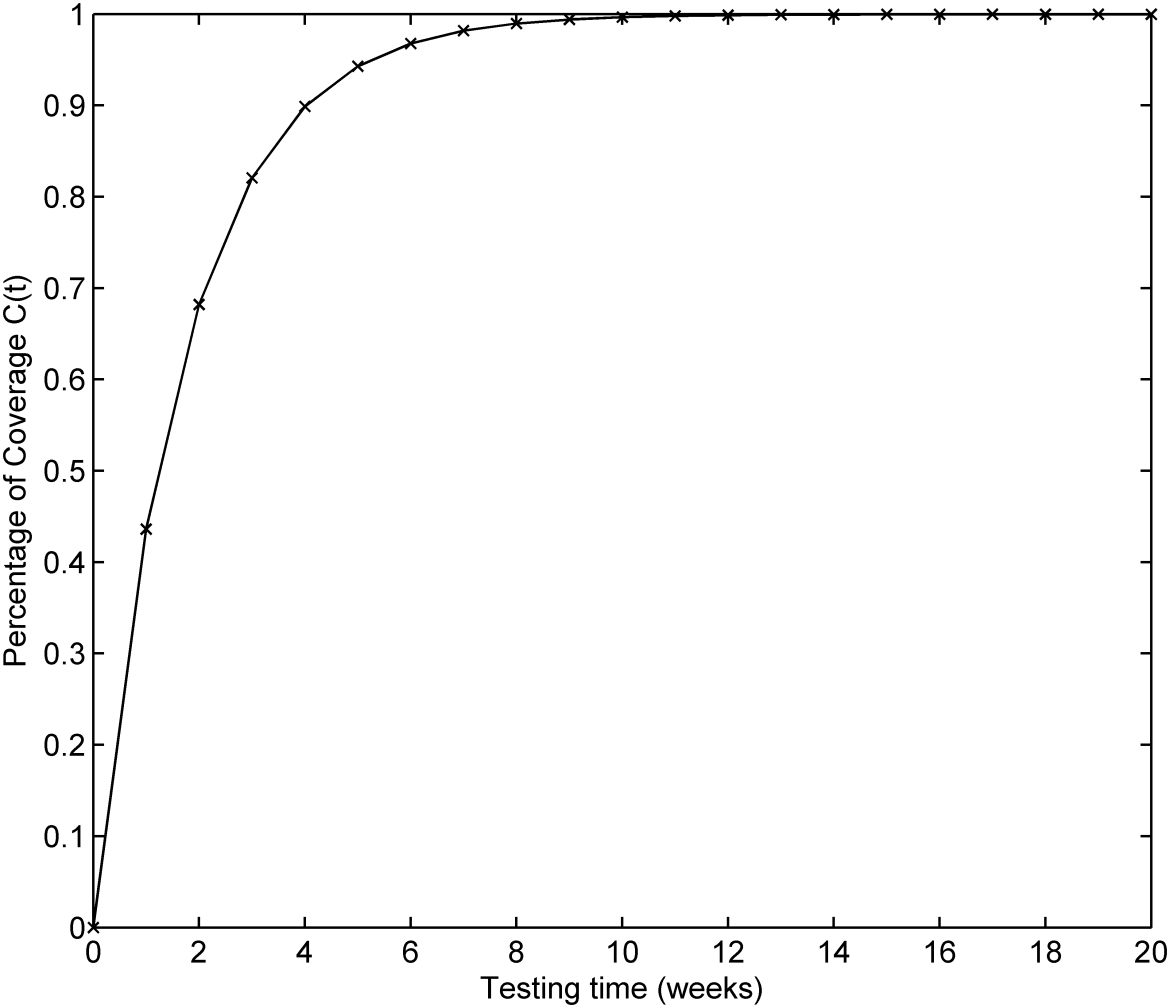
Testing coverage of DS-2.

**Fig 5:**
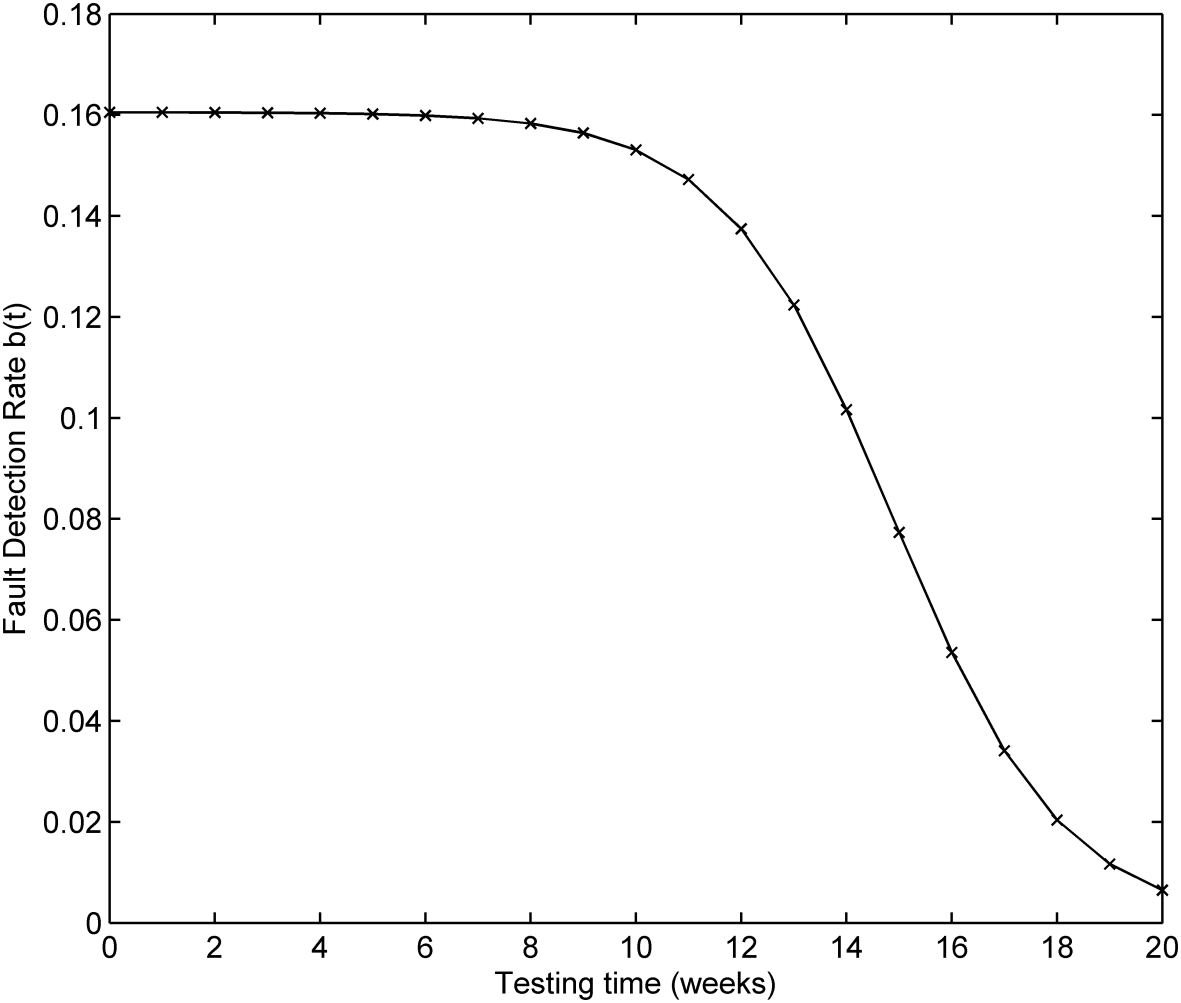
Fault detection rate of DS-2.

**Fig 6:**
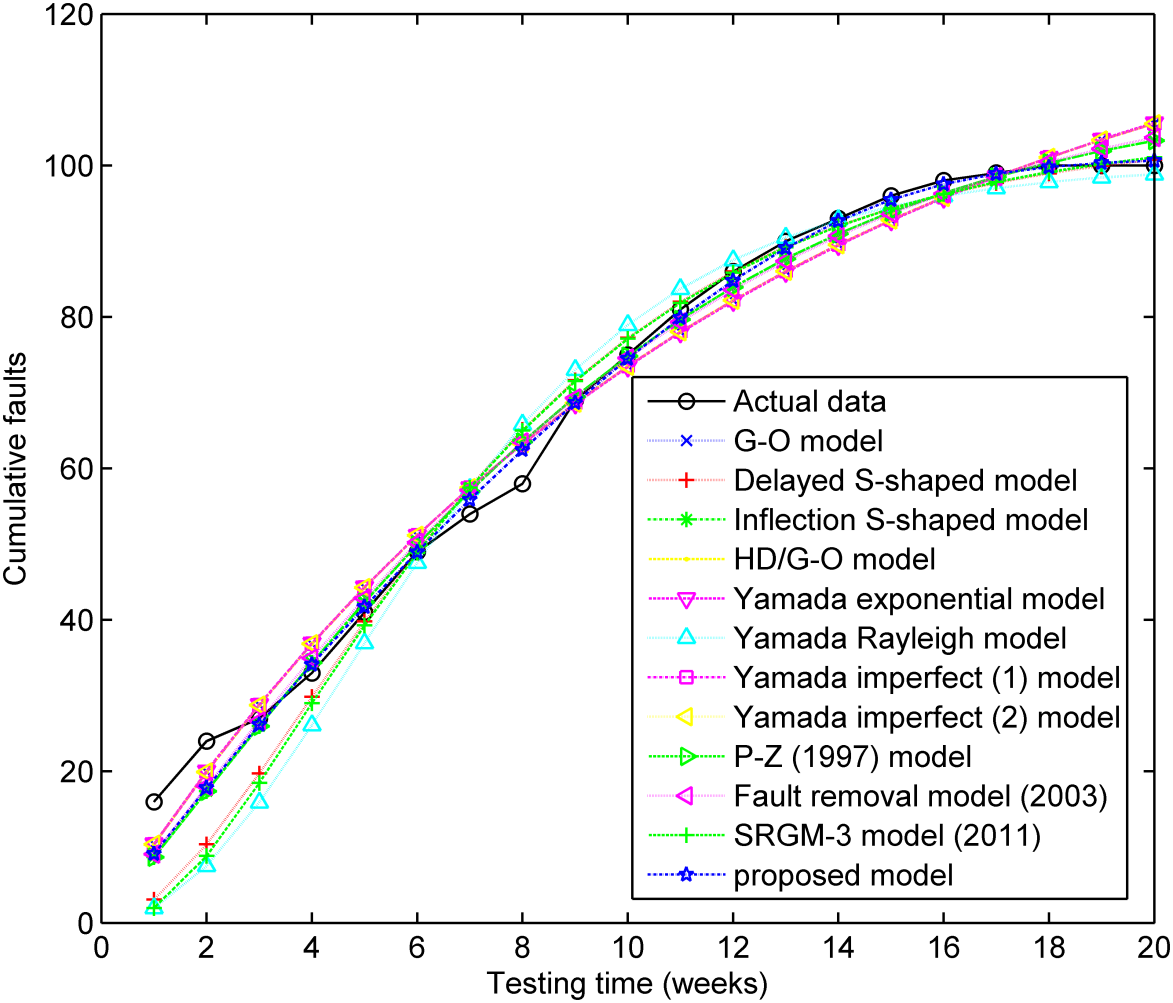
The fitting results of SRGMs compared with actual data for DS-2.

The testing coverage function of the proposed model based on the parameters estimated by LSE according to DS-2 is graphically illustrated in Fig 4, the changing trend of the testing coverage over time also shows the aforementioned trend. Fig 5 depicts the fault detection rate over time of DS-2. It shows that the fault detection rate displays a non-increasing S-shaped trend with different decreasing rate at different phase, firstly its decreasing rate changes from flat to more, then the fault detection rate decreases from fast to slow. The fitting comparison of all models for DS-2 is graphically illustrated in Fig 6. It can be seen that the proposed model fits the actual data better than all other models.

### PL/I database application

In this section, we examine models using another data cited from Ohba (Data Set 3, DS-3) [5], which has also been widely used in many studies, such as [22,45,46]. The failure data are given in Table 6 with time unit week. There are totally 328 faults detected within about 19 weeks. All data points are used to fit the models and estimate the models’ parameters.

**Table 6:** Failure data of DS-3.

Testing time (weeks)	Cumulative faults	Testing time (weeks)	Cumulative faults
1	15	11	233
2	44	12	255
3	66	13	276
4	103	14	298
5	105	15	304
6	110	16	311
7	146	17	320
8	175	18	325
9	179	19	328
10	206		

Here we only use MLE to estimate the models’ parameters recorded in Table 7. MSE, *R*^2^, Adjusted *R*^2^ and AIC values are all calculated on the parameters obtained by MLE.

**Table 7:** Comparison of SRGMs’ descriptive power for DS-3.

Model No.	Model Name	MLE Method				
		Model Parameter Estimation Results	MSE	*R*^2^	Adjusted_ *R*^2^	AIC
1	G-O model	$\hat{a}=513.1$ $\hat{b}=0.05365$	248.2227	0.9785	0.9758	220.7602
2	Delayed S-shaped model	$\hat{a}=359.9$ $\hat{b}=0.2126$	211.0699	0.9817	0.9794	222.3754
3	Inflection S-shaped model	$\hat{a}=355.1$ $\hat{b}=0.2129$ $\hat{\beta }=3.629$	114.7818	0.9906	0.9888	205.0108
4	HD/G-O model	$\hat{a}=\text{513}.1$ $\hat{b}=\text{0}.05365$ $\hat{c}=\text{0}.08779$	263.7371	0.9785	0.9742	222.7602
5	Yamada exponential model	$\hat{a}=\text{826}.7$ $\hat{\beta }=0.02387$ $\hat{\gamma }=\text{0}.2190$ $\hat{\alpha }=\text{6}.332$	287.1442	0.9780	0.9718	226.1432
6	Yamada Rayleigh model	$\hat{a}=469.4$ $\hat{\beta }=0.01403$ $\hat{\gamma }=0.3685$ $\hat{\alpha }=3.538$	346.2307	0.9735	0.9660	247.8212
7	Yamada imperfect 1 model	$\hat{a}=513.1$ $\hat{b}=0.05365$ $\hat{\alpha }=1.0\text{e-10}$	263.7372	0.9785	0.9742	222.7602
8	Yamada imperfect 2 mode	$\hat{a}=513.1$ $\hat{b}=0.05365$ $\hat{\alpha }=1.0\text{e-10}$	263.7366	0.9785	0.9742	222.7602
9	P-Z model	$\hat{a}=1.0\text{e-10}$ $\hat{b}=0.2129$ $\hat{c}=355.1$ $\hat{\alpha }=\text{5}.634\text{e}-05$ $\hat{\beta }=3.629$	131.1790	0.9906	0.9870	209.0108
10	Fault removal model	$\hat{a}=321.9$ $\hat{\alpha }=100.0$ $\hat{b}=0.1312$ $\hat{p}=0.9836$ $\hat{c}=5.403$ $\hat{\beta }=\text{0}.01765$	110.8502	0.9927	0.9890	204.6694
11	SRGM-3 model	$\hat{A}=372.7$ $\hat{\alpha }=0.1408$ $\hat{b}=5.108$ $\hat{p}=0.01854$	215.7341	0.9835	0.9788	215.9364
12	proposed model	$\hat{a}=628.2$ $\hat{\alpha }=0.5$ $\hat{c}=0.1781$ $\hat{p}=0.6$ $\hat{\beta }=0.058$ $\hat{\gamma }=0.6193$ $\hat{\text{A}}=0.9999$	**93.8861**	**0.9943**	**0.9906**	**203.3359**

Notes: The bold numbers mean the result of the best SRGM in this column.

It can be seen that compared to all models using the MSE, *R*^2^, Adjusted *R*^2^ and AIC criteria, the proposed model displays the smallest MSE value and AIC value, the largest *R*^2^ value and Adjusted_*R*^2^ value, the values are 93.8861, 203.3359, 0.9943 and 0.9906, respectively. That is to say, we can deduce that the proposed model performs the best among all models in goodness-of-fit behavior.

Moreover, some additional information can be acquired from the estimation values of the parameters given by the proposed model. For instance, the fault removal efficiency is 60%, which is below the average value according to [20], indicating the skill of the testing team should be improved. The initial fault content is estimated 628, and the fault introduction rate is 0.5, the expected total number of faults detected is 792 at 19 weeks. Thus, at 19 weeks, which is the assumed stopping time point, there are still about 464 remaining faults present in the software. This means that many faults still remain in the software at the end of the testing phase. The fault introduction probability is 0.5, i.e., on average, 1 fault will be introduced when 2 faults are removed on average. So more testing training should be given to the testers and their testing skill should be improved greatly.

**Fig 7:**
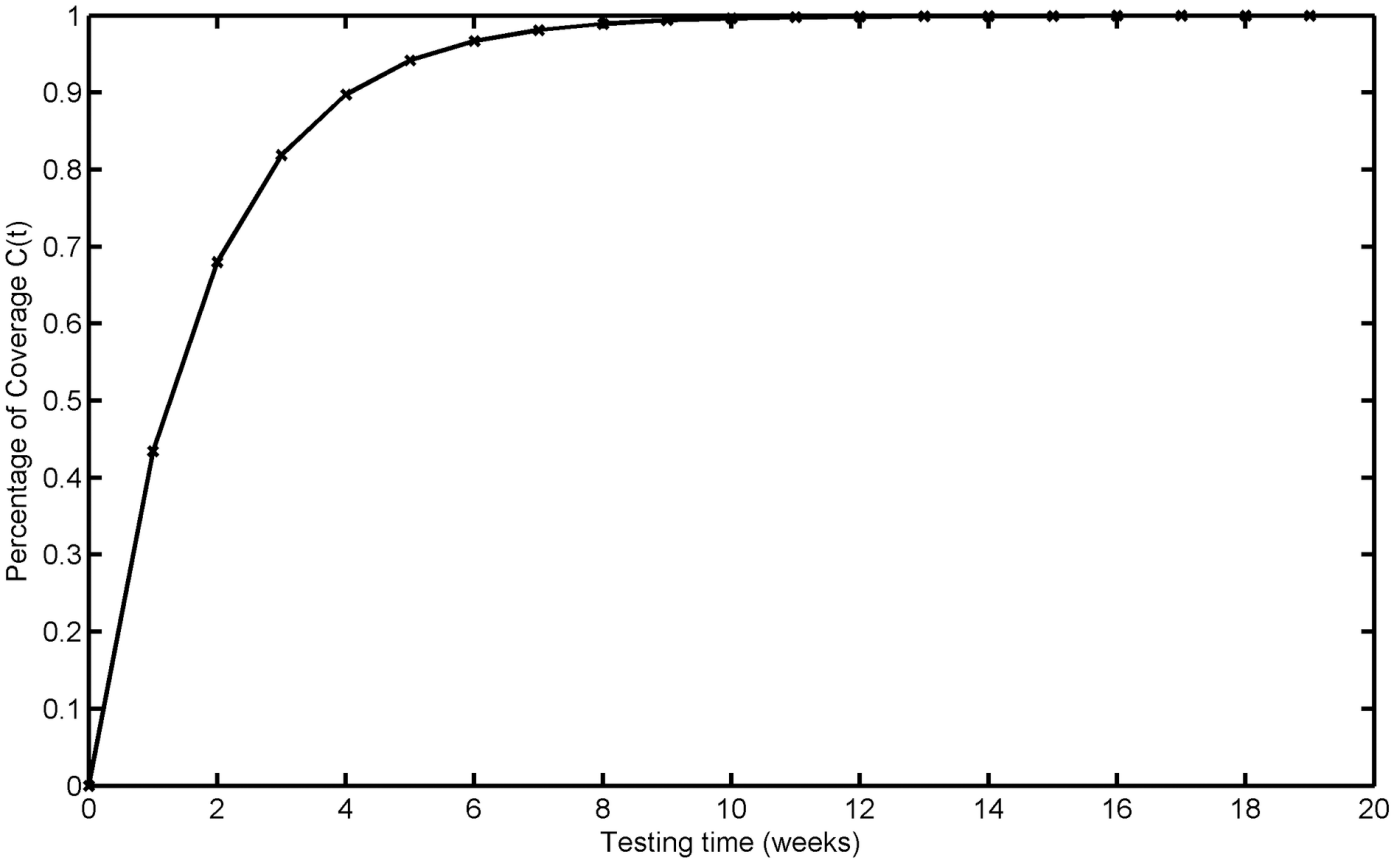
Testing coverage of DS-3.

**Fig 8:**
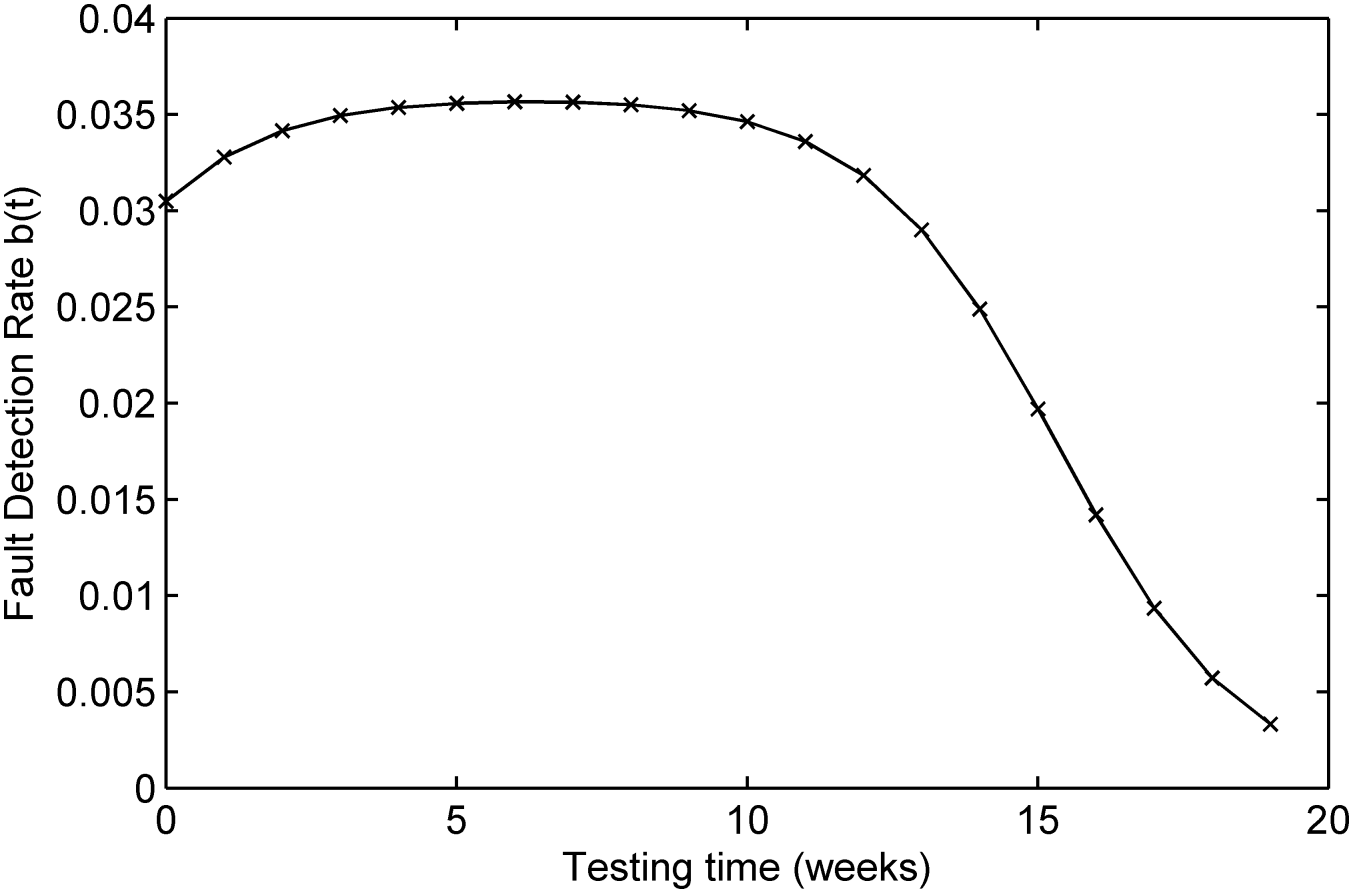
Fault detection rate of DS-3.

**Fig 9:**
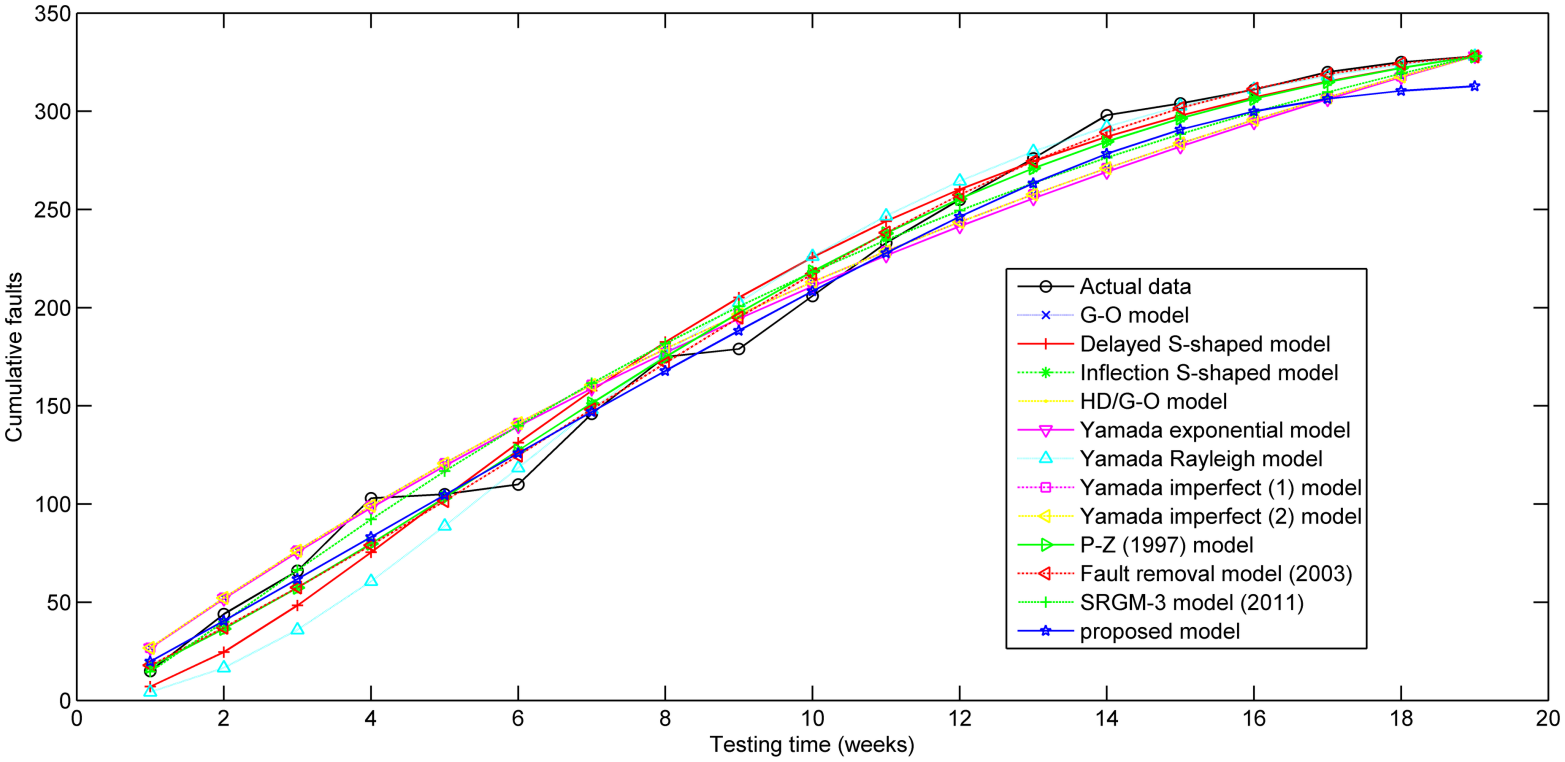
The fitting results of SRGMs compared with actual data for DS-3.

The testing coverage function of the proposed model based on the parameters estimated by MLE according to DS-3 is graphically illustrated in Fig 7, the changing trend of the testing coverage over time also shows the aforementioned trend. Fig 8 depicts the fault detection rate over time of DS-3. It shows that the fault detection rate displays an S-shaped trend firstly increasing and then decreasing with different decreasing rate at different phase, e.g. firstly its decreasing rate changes from flat to more, then the fault detection rate decreases from fast to slow. The fitting comparison of all models for DS-3 is graphically illustrated in Fig 9. It can be seen that the proposed model fits the actual data better than all other models.

### Comparison of models’ predictive power

In order to validate the performance of the proposed model’s predictive power, we divide the above three data sets into two parts. For DS-1, we use the first 80% of data points to estimate the models’ parameters, then use the remaining data to compare the models’ predictive power. Table 8 gives both SSE values in the context of LSE and AIC values in the context of MLE, respectively. It shows that the proposed model provides the lowest value of SSE (533.3269) and the smallest value of AIC (599.7586), which are both far less than other models’ values. For example, the proposed model is followed by the inflection S-shaped model with an SSE value of 2774.2 and the P-Z model with an SSE of 2814.9, which are approximately 5 times as large as that of the proposed model. Meanwhile, the Yamada exponential model displays an AIC value of 685.9234 and the Yamada imperfect 1 model gives an AIC of 683.8968, which are approximately 1.15 times as large as that of the proposed model.

**Table 8:** Comparison of SRGMs’ predictive power for DS-1, DS-2 and DS-3.

Model No.	Model Name	80% OF DS-1		90% OF DS-2		95% OF DS-3	
		LSE	MLE	LSE	MLE	MLE	MLE
		SSE(prediction)	AIC	SSE(prediction)	SSE(prediction)	SSE(prediction)	AIC
1	G-O model	6.7713e+04	681.8968	127.7521	19.6070	73.0257	209.8042
2	Delayed S-shaped model	3.5343e+03	612.0752	3.5409	3.5409	10.7484	217.4616
3	Inflection S-shaped model	2.7742e+03	607.7088	69.2427	11.8312	13.9427	199.8602
4	HD/G-O model	6.7713e+04	683.8968	127.7521	19.6070	73.0261	211.8042
5	Yamada exponential model	6.0381e+04	685.9234	129.9145	20.3747	67.0656	213.8376
6	Yamada Rayleigh model	3.4667e+05	631.7	8.3464	1.6944	3.7106	244.3408
7	Yamada imperfect 1 model	6.7713e+04	683.8968	127.7523	19.6065	73.0260	211.8042
8	Yamada imperfect 2 model	5.7234e+04	683.8968	127.7522	19.6074	73.0266	211.8042
9	P-Z model	2.8149e+03	611.6886	69.2427	11.8312	13.9429	203.8602
10	Fault removal model	1.9332e+04	618.0582	38.6916	19.6069	1.0408	201.4796
11	SRGM-3 model	1.1495e+04	630.9322	4.4774e+03	859.1574	49.2648	207.0564
12	proposed model	**533.3269**	**599.7586**	**2.6775**	**0.7418**	**0.2231**	**199.7570**

Notes: The bold numbers mean the results of the best SRGM in this column.

For DS-2, we use the first 90% of data points to estimate the models’ parameters, then use the remaining data to compare the models’ predictive power. Here we also use both LSE and MLE methods to estimate the models’ parameters. In terms of SSE values listed in Table 8, the proposed model still offers the smallest values of SSE at 2.6775 and 0.7418, though followed by the delayed S-shaped model with an SSE of 3.5409 and the Yamada Rayleigh model with an SSE of 1.6944, which are only 2 times as large as that of the proposed model, but other models have very larger SSE values from 3 times to 1672 times as large as that of the proposed one.

For DS-3, we use the first 95% of data points to estimate the models’ parameters, then use the remaining data to compare the models’ predictive power. Here we only use MLE method to estimate the models’ parameters. From Table 8, it can be seen that the proposed model provides both the lowest value of SSE (0.2231) and the smallest value of AIC (199.7570).

Therefore, according to these results listed in Table 8, we can conclude that the proposed model provides better prediction performance.

### Sensitivity analysis

**Fig 10:**
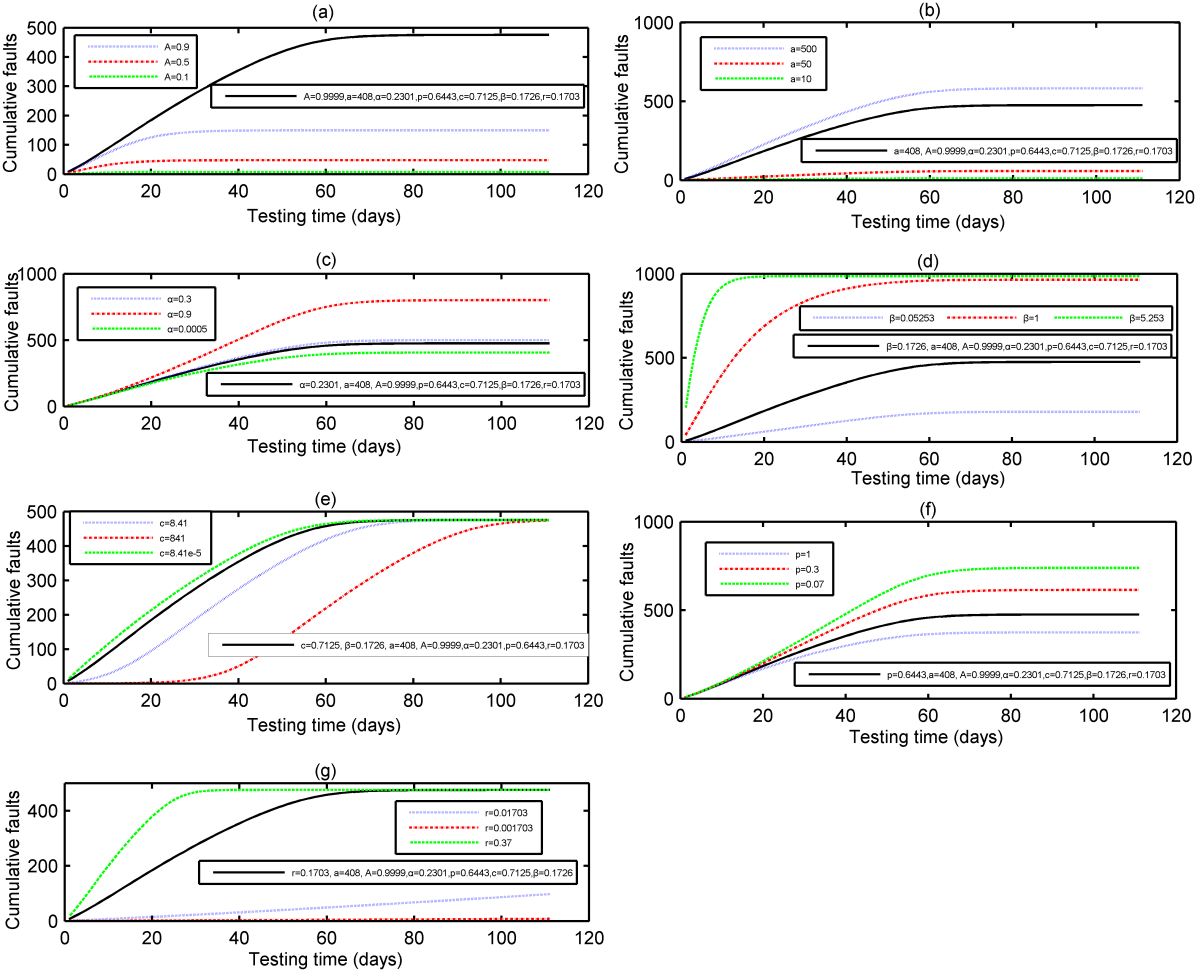
The sensitivity analysis of the parameters of the proposed model for DS-1. (a) Dependence of Parameter A (DS-1) (b) Dependence of Parameter a (DS-1) (c) Dependence of Parameter α (DS-1) (d) Dependence of Parameter β (DS-1) (e) Dependence of Parameter c (DS-1) (f) Dependence of Parameter p (DS-1) (g) Dependence of Parameter r (DS-1).

**Fig 11:**
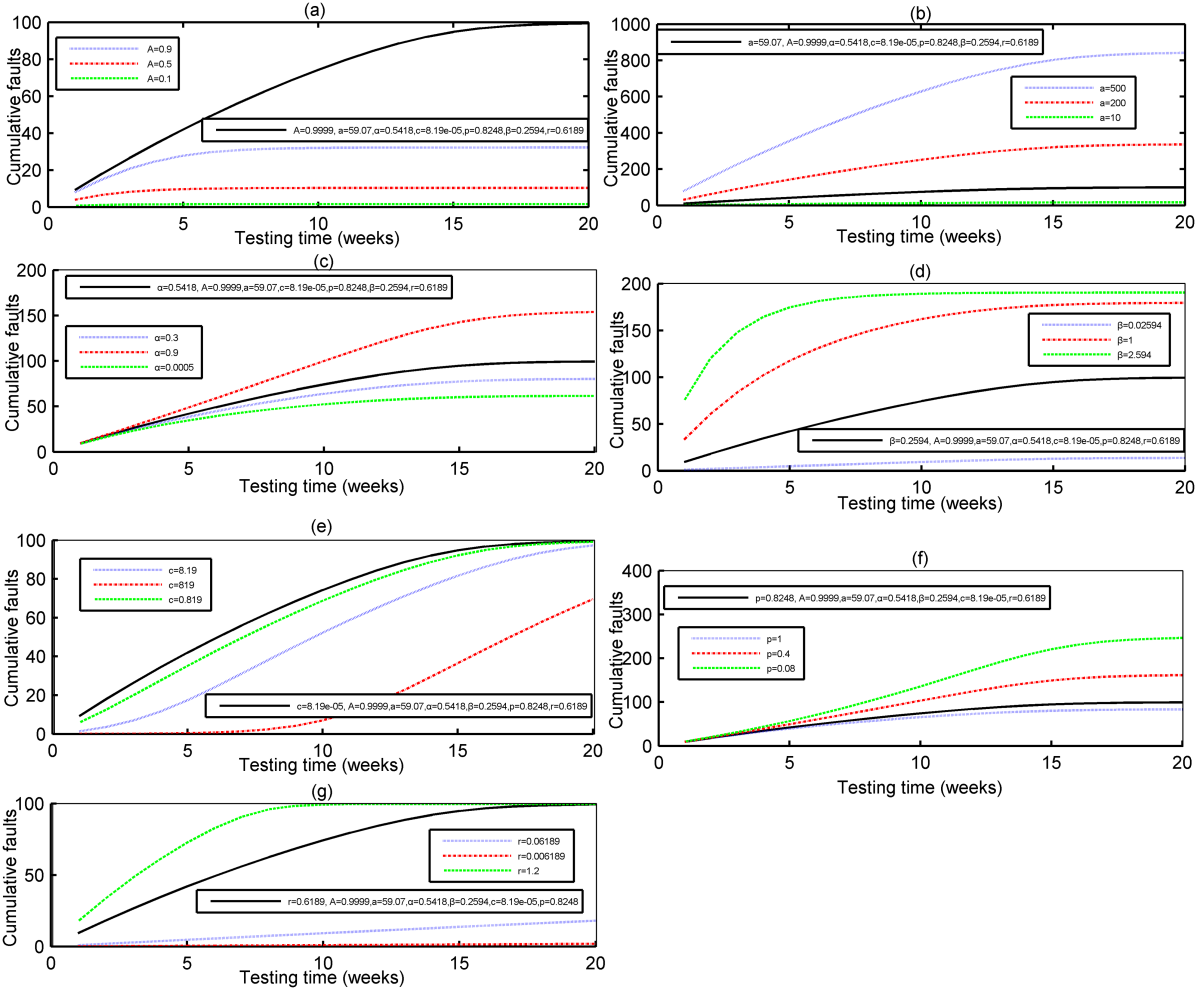
The sensitivity analysis of the parameters of the proposed model for DS-2. (a) Dependence of Parameter A (DS-2) (b) Dependence of Parameter a (DS-2) (c) Dependence of Parameter α (DS-2) (d) Dependence of Parameter β (DS-2) (e) Dependence of Parameter c (DS-2) (f) Dependence of Parameter p (DS-2) (g) Dependence of Parameter r (DS-2).

Here we conduct the sensitivity analysis to study each parameter’s impact on the robustness of the proposed model, in which one parameter is changeable, while the other parameters are set to their fixed values. Due to limited space, here we only give the results based on DS-1 and DS-2, the same conclusion can be obtained on DS-3, too. Fig 10(a)–10(g) show the sensitivity of parameters *A*, *α*, *α*, *β*, *p*, *c* and *r* in the proposed model based on DS-1 respectively. From Fig 10(a)–10(g), it can be seen that the cumulative number of detected faults will be apparently changed with the expected number of initial fault number *α*, fault introduction rate *α*, the fault removal efficiency *p*, the probability constant *β*, the maximum testing coverage percentage *A*, scale parameter *c* and shape parameter *r* changing accordingly. Thus, parameters *A*, *α*, *α*, *β*, *p*, *c* and *r* are all influential parameters in the proposed model. Fig 11(a)–11(g) also show the similar results for DS-2. So these parameters can be regarded as influential parameters in the proposed model.

## Conclusions

In this paper, an imperfect debugging model NHPP-based is developed to incorporate both error generation and imperfect fault removal efficiency, together with considering inflected S-shaped testing coverage to denote the fault detection rate function. Comparisons of this model with several other existing NHPP models have been presented in detail. In addition, three widely used failure data sets are provided for validating the goodness-of-fit and predictive performance of the proposed model. Moreover, five comparison criteria are used to evaluate model performance and the results conclude that the proposed model can fit and predict better. Thus, the results obtaining from the proposed model are encouraging. Furthermore, the sensitivity analysis displays that parameters *A*, *α*, *α*, *β*, *p*, *c* and *r* are influential parameters in the proposed model.

The limitations for the proposed model are analyzed as follows:

1. In our experiments, quantity and kind of fault data sets seem to be limited. To be well-known, using more data sets and more kinds of data sets can give more effective demonstration for the model’s performance. However, we used only three real-world data sets to validate the model’s performance. Thus, fault data sets in quantity and more kinds are needed in the future work to give a more in-depth validation.
2. The proposed model assumes the fault removal efficiency to be a constant to simplify the model’s mathematical derivation and calculation. But in practical software debugging process, the fault removal efficiency may be changed dependent on time because it depends on many factors such as the skill of the testing team, the complexity of software system, the testing strategy and the testing environment etc. Thus, the fault removal efficiency may take some kinds of complicated function forms, e.g., a time dependent function rather than a constant. Therefore, more forms of fault removal efficiency function should be studied in the future research.

## References

[1] Lyu MR. Handbook of Software Reliability Engineering. New York: McGraw-Hill; 1996.

[2] Wood A, Predicting Software Reliability. IEEE Computer; 1996(11):69–77.

[3] Goel AL, Okumoto K. Time-dependent error-detection rate model for software reliability and other performance measures. IEEE Trans Reliab. 1980;(3):206–11.

[4] Misra PN. Software reliability analysis. Ibm Systems Journal. 1983;22(3):262–70.

[5] Ohba M. Software Reliability Analysis Models. Ibm Journal of Research & Development. 1984;21(4):428–43.

[6] Yamada S, Ohtera H, Ohba M. Testing-domain dependent software reliability models. Computers & Mathematics with Applications. 1992;24(1–2):79–86.

[7] Pham H. Software Reliability: John Wiley & Sons, Inc; 1999.

[8] Ohba M. Inflection S-Shaped Software Reliability Growth Model Stochastic Models in Reliability Theory. Berlin: Springer,1984 144–62

[9] Jelinski Z, Moranda P. SOFTWARE RELIABILITY RESEARCH Statistical Computer Performance Evaluation. 1972:465–84.

[10] Yamada S, Ohba M, Osaki S. S-Shaped Reliability Growth Modeling for Software Error Detection. IEEE Transactions on Reliability. 1983;32(5):475–84.

[11] Goel AL. Software Reliability Models: Assumptions, Limitations, and Applicability. Software Engineering IEEE Transactions on. 1985;SE-11(12):1411–23.

[12] Kareer N, Kapur PK, Grover PS. An S-shaped software reliability growth model with two types of errors. Microelectronics Reliability. 1990;30(6):1085–90.

[13] Pham H. Software reliability assessment: Imperfect debugging and multiple fault types in software development EG&G-RAAM-10737, Idaho National Laboratory, 1993.

[14] Lo JH, Huang CY. An integration of fault detection and correction processes in software reliability analysis. Journal of Systems & Software. 2006;79(9):1312–23.

[15] Xie M, Hu QP, Wu YP, Ng SH. A study of the modeling and analysis of software fault-detection and fault-correction processes. Quality & Reliability Engineering. 2006;23(4):459–70.

[16] YAMADA S, TOKUNO K, OSAKI S. Imperfect debugging models with fault introduction rate for software reliability assessment. Int. J. Syst. Sci. 1992;23(12):2241–52.

[17] Ohba M, Chou XM. Does Imperfect Debugging Affect Software Reliability Growth? International Conference on Software Engineering; 1989:237–244.

[18] Pham H, Zhang X. An NHPP Software Reliability Model and Its Comparison. International Journal of Reliability Quality & Safety Engineering. 1997;14(3):269–82.

[19] Pham H, Nordmann L, Zhang Z. A general imperfect-software-debugging model with S-shaped fault-detection rate. IEEE Transactions on Reliability. 1999;48(2):169–75.

[20] Jones C. Software defect-removal efficiency. Computer. 1996;29(4):94–5.

[21] Zhang X, Teng X, Pham H. Considering fault removal efficiency in software reliability assessment. Systems Man & Cybernetics Part A Systems & Humans IEEE Transactions on. 2003;33(1):114–20.

[22] Kapur PK, Pham H, Anand S, Yadav K. A Unified Approach for Developing Software Reliability Growth Models in the Presence of Imperfect Debugging and Error Generation. Reliability IEEE Transactions on. 2011;60(1):331–40.

[23] Goel AL, Okumoto K, editors. A Markovian model for reliability and other performance measures of software systems. International Workshop on Managing Requirements Knowledge; 1979.

[24] Kremer W. Birth-Death and Bug Counting. Reliability IEEE Transactions on. 1983;R-32(1):37–47.

[25] Wang L, Hu Q, Liu J. Software reliability growth modeling and analysis with dual fault detection and correction processes. IIE Transactions. 2015;48(4):359–70. doi: 10.1080/0740817x.2015.1096432

[26] Wang J, Wu Z, Shu Y, Zhang Z. An optimized method for software reliability model based on nonhomogeneous Poisson process. Applied Mathematical Modelling. 2016;40(13–14):6324–39. doi: 10.1016/j.apm.2016.01.016

[27] Wang J, Wu Z. Study of the nonlinear imperfect software debugging model. Reliability Engineering & System Safety. 2016;153:180–92. doi: 10.1016/j.ress.2016.05.003

[28] Wang J, Wu Z, Shu Y, Zhang Z. An imperfect software debugging model considering log-logistic distribution fault content function. Journal of Systems and Software. 2015;100:167–81. doi: 10.1016/j.jss.2014.10.040

[29] Pham H. A new software reliability model with Vtub-shaped fault-detection rate and the uncertainty of operating environments. Optimization. 2014;63(10):1481–90. doi: 10.1080/02331934.2013.854787

[30] Peng R, Li YF, Zhang WJ, Hu QP. Testing effort dependent software reliability model for imperfect debugging process considering both detection and correction. Reliability Engineering & System Safety. 2014;126:37–43. doi: 10.1016/j.ress.2014.01.004

[31] Huang CY, Kuo SY, Lyu MR. An Assessment of Testing-Effort Dependent Software Reliability Growth Models. Reliability IEEE Transactions on. 2007;56(2):198–211.

[32] Shibata K, Rinsaka K, Dohi T. Metrics-Based Software Reliability Models Using Non-homogeneous Poisson Processes. International Symposium on Software Reliability Engineering; 2006:IEEE; 2006:52–61.

[33] Cai X, Lyu MR. Software Reliability Modeling with Test Coverage: Experimentation and Measurement with A Fault-Tolerant Software Project. International Symposium on Software Reliability Engineering; 2007:IEEE;2007:17–26.

[34] Malaiya YK, Li MN, Bieman JM, Karcich R. Software reliability growth with test coverage. IEEE Transactions on Reliability. 2002;51(4):420–6.

[35] Pham H, Zhang X. NHPP software reliability and cost models with testing coverage. European Journal of Operational Research. 2003;145(2):443–54.

[36] Vouk MA. Using Reliability Models During Testing With Non-Operational Profiles. Proc. 2nd Bellcore/Purdue Workshop on Issues in Software Reliability Estimation;1992:103–11.

[37] Gokhale SS, Trivedi KS. A time/structure based software reliability model. Annals of Software Engineering. 1999;8(1):85–121.

[38] Park JY, Lee G, Park JH. A class of coverage growth functions and its practical application. Journal of the Korean Statistical Society. 2008;37(3):241–7.

[39] Hossain SA, Dahiya RC. Estimating the parameters of a non-homogeneous Poisson-process model for software reliability. IEEE Transactions on Reliability. 1994;42(4):604–12.

[40] Zhao M., Xie M. On Maximum Likelihood Estimation for a General Non-homogeneous Poisson Process. Scandinavian Journal of Statistics, 1996, 23(4):597–607.

[41] Huang CY, Lyu MR. Estimation and Analysis of Some Generalized Multiple Change-Point Software Reliability Models. IEEE Transactions on Reliability. 2011;60(2):498–514.

[42] Zhang X, Pham H. Comparisons of nonhomogeneous Poisson process software reliability models and its applications. International Journal of Systems Science. 2000;31(9):1115–23. doi: 10.1080/002077200418397

[43] Tohma Y, Yamano H, Ohba M, Jacoby R. The estimation of parameters of the hypergeometric distribution and its application to the software reliability growth model. IEEE Transactions on Software Engineering. 1991;17(5):483–9.

[44] Xu J, Yao S. Software Reliability Growth Model with Partial Differential Equation for Various Debugging Processes. Mathematical Problems in Engineering. 2016;2016:1–13. doi: 10.1155/2016/2476584

[45] Huang CY, Lin CT, Kuo SY, Lyu MR, Sue CC. Software Reliability Growth Models Incorporating Fault Dependency with Various Debugging Time Lags. Computer Software and Applications Conference, 2004. COMPSAC 2004. Proceedings of the, International. IEEE Xplore, 2004:186–191 vol.1.

[46] Zhang J, Lu Y, Yang S, Xu C. NHPP-based software reliability model considering testing effort and multivariate fault detection rate. Journal of systems engineering and electronics, 2016, 27(1):260–270.

